# Contribution of Gray Matter Atrophy and White Matter Damage to Cognitive Impairment in Mildly Disabled Relapsing-Remitting Multiple Sclerosis Patients

**DOI:** 10.3390/diagnostics11030578

**Published:** 2021-03-23

**Authors:** Ángela Bernabéu-Sanz, Sandra Morales, Valery Naranjo, Ángel P. Sempere

**Affiliations:** 1 Magnetic Resonance Department, Hospital General Universitario de Alicante, Inscanner SL, 03010 Alicante, Spain; 2 Instituto de Investigación e Innovación en Bioingeniería, I3B, Universitat Politècnica de València, 46022 Valencia, Spain; sanmomar@i3b.upv.es (S.M.); vnaranjo@upv.es (V.N.); 3 Neurology Service, Hospital General Universitario de Alicante, 03010 Alicante, Spain; aperezs@mac.com; 4 Department of Clinical Medicine, Miguel Hernández University, 03202 Alicante, Spain; 5 The Alicante Institute for Health and Biomedical Research (ISABIAL), Hospital General Universitario de Alicante, 03010 Alicante, Spain

**Keywords:** relapsing-remitting multiple sclerosis, cognitive impairment, diffusion tensor imaging, thalami atrophy, white matter damage

## Abstract

Cognitive impairment (CI) is frequently present in multiple sclerosis patients. Despite ongoing research, the neurological substrates have not been fully elucidated. In this study we investigated the contribution of gray and white matter in the CI observed in mildly disabled relapsing-remitting multiple sclerosis (RRMS) patients. For that purpose, 30 patients with RRMS (median EDSS = 2), and 30 age- and sex-matched healthy controls were studied. CI was assessed using the symbol digit modalities test (SDMT) and the memory alteration test. Brain magnetic resonance imaging, diffusion tensor imaging (DTI), voxel-based morphometry (VBM), brain segmentation, thalamic vertex analysis, and connectivity-based thalamic parcellation analyses were performed. RRMS patients scored significantly lower in both cognitive tests. In the patient group, significant atrophy in the thalami was observed. Multiple regression analyses revealed associations between SDMT scores and GM volume in both hemispheres in the temporal, parietal, frontal, and occipital lobes. The DTI results pointed to white matter damage in all thalamocortical connections, the corpus callosum, and several fasciculi. Multiple regression and correlation analyses suggested that in RRMS patients with mild disease, thalamic atrophy and thalamocortical connection damage may lead to slower cognitive processing. Furthermore, white matter damage at specific fasciculi may be related to episodic memory impairment.

## 1. Introduction

Multiple sclerosis (MS) is the most prevalent chronic inflammatory disease of the central nervous system. While its course is characterized by a wide range of clinical manifestations, cognitive impairment (CI) is a frequent and important symptom, occurring in 40–70% of patients [1]. In MS, CI starts early in the disease course and generally worsens over time. It is influenced by many factors such as genetics, sex, intelligence, disease course, and health-promoting behaviors [2]; and may differ significantly between progressive vs. relapsing disease [3]. Slowed cognitive processing and episodic memory decline are the most common cognitive deficits in MS, alongside difficulties in executive function, verbal fluency, and visuospatial analysis [4,5].

Despite ongoing research, the neurological substrates of CI in MS have not been fully elucidated, but may involve the atrophy of both white matter (WM) and gray matter (GM), with GM atrophy predominating in the early stages, followed by WM atrophy [6]. Recent studies have suggested a main role of the thalamus in CI, with several studies reporting decreased neuronal integrity and macroscopic thalamic atrophy even in the early stages of the disease [7]. Thalamic atrophy is one of the earliest and most prominent signs of MS, with progressive atrophy detected in all MS types [8]. It is associated with CI and the progression of disability, and its detection may aid in the identification of patients with MS at greater risk of cognitive decline [9]. Indeed, thalamic volume has been suggested as a potential biomarker for CI detection, as it can be relatively easily measured [10,11].

However, determining if thalamic atrophy is the main cause of CI, or if there are other brain structures involved warrants further research. Given the multifocal WM pathology observed in MS, the CI could also be caused by disconnections between cognitively important cortical and subcortical regions. Recent MS studies have found microstructural abnormalities in specific WM tracts, such as the cingulum, corpus callosum, superior and middle cerebellar peduncles, and uncinate fasciculus, which are associated with cognitive performance [12,13,14]. These observations suggest that the CI observed in MS patients might be caused by a multiple disconnection syndrome [15].

In fact, thalamic pathological mechanisms cannot be assessed in isolation. As a brain structure with widespread cortical and subcortical connections, the thalamic atrophy observed in MS may be a retrograde event caused by axonal transection in WM tracts projecting from the thalamus, or it may be secondary to transsynaptic deafferentation of thalamic neurons. Since the thalamus is very sensitive to retrograde degeneration [16], more studies are needed to determine the exact link between the pathologic processes operating in the subcortical GM with respect to those in the WM and their association with CI. In this sense, Henry et al. pointed out a common mechanism of thalamic injury, where lesions cause distal WM injury, leading directly to the loss of thalamic neurons and volume [17]. In a recent follow-up study, Weeda et al. found a direct association between thalamocortical connection damage and thalamic atrophy, suggesting a directionality pattern [18].

Based on this knowledge, the present study investigated whether the CI observed in mildly disabled relapsing-remitting multiple sclerosis (RRMS) patients might be caused by a combined loss of white matter integrity and gray matter atrophy, both in specific brain areas. We hypothesized that the observed CI is a result, not only of thalamic atrophy, but also of the disruption of critical white matter tracts, leading to reduced functional connectivity between cortical and subcortical regions, and resulting in impairment in specific cognitive domains.

## 2. Materials and Methods

### 2.1. Participants

Thirty consecutive patients fulfilling the 2010 McDonald criteria for MS [19] and 30 healthy age- and sex-matched controls (HC) were included in the study. Patients with MS were recruited at the MS Clinic of the University Hospital of Alicante.

Controls were recruited from the same social and cultural background as the patients. Inclusion criteria for patients with MS were: (1) diagnosis of RRMS [20], (2) aged 18–65 years, and (3) scores on the expanded disability status scale (EDSS) from 0 to 3 [21].

The suitability of all participants was decided based on clinical history, cognitive assessment, and physical examination. Exclusion criteria included claustrophobia, metal implants, pregnancy, a history of any medical or psychiatric disorder that could affect cognitive function, alcohol or drug abuse, relapse, or corticosteroid treatment within the previous three months.

All participants underwent a physical and neurological examination by an experienced neurologist (APS). The EDSS was assessed in patients with MS by the same neurologist. Cognitive performance was assessed using the symbol digit modalities test (SDMT) [22] and the memory alteration test (M@T) [23]. The M@T is a verbal episodic and semantic memory test that has been validated in primary care populations in Spain [24]. Physical and cognitive assessments were performed within the same week as the MRI. Cognitive test scores were converted into Z-scores, as described previously [25].

### 2.2. Magnetic Resonance Imaging

Scans were acquired using an Achieva 3T series-X MRI system (Philips Medical, The Netherlands) with a SENSE-Neurovascular coil provided by the manufacturer. The protocol included axial brain 3-mm-thick slices, 0-mm inter-slice interval: T1-weighted (TE/TR = 10 ms/558 ms), T2-TSE (TE/TR = 80 ms/3760 ms), and T2-FLAIR (TE/TR = 125 ms/11 s). FLAIR-3D-TSE (TR/TE/TI = 4800 ms/365 ms/1650 ms, voxel dimensions = 1 × 1 × 1.2 mm). Axial high-resolution 3D-T1-weighted gradient-echo scans (160 slices, 1-mm isotropic voxels, field of view 250 × 250 mm, TR = 13 ms, and TE = 7.3 ms). Diffusion tensor imaging (DTI) was performed with a transverse slice orientation using a single-shot EPI sequence with diffusion encoding in 32 directions (values 0 and 800 s/mm^2^), 60 slices, voxel size 2 × 2 × 2 mm, and a SENSE factor of 1.9. No sedation was administered during the study.

All MRI images were checked to verify their quality and the absence of gross anatomical malformations or artifacts (e.g., head coverage, radiofrequency noise, signal inhomogeneity and/or susceptibility, motion artifacts, or metal-induced susceptibility artifacts).

### 2.3. Brain Segmentation and Volumetry

For volumetric measurements, a high-resolution T1-weighted gradient-echo scan was used. All scans were analyzed using BRAIM software, as described previously [26]. After skull stripping, the WM, GM, and cerebrospinal fluid (CSF) were automatically segmented. The automatic segmentation results were saved, and semi-automatic segmentation was performed for the thalamus, hippocampus, caudate nucleus, and putamen. Volume data were then obtained from the segmented structures. Subcortical structures were normalized to the intracranial volume (ICV). ICV was calculated as the sum of WM, GM, and CSF volumes. The brain parenchymal fraction (BPF) was calculated for each participant by dividing the sum of all GM and WM fractions by the ICV.

To calculate the total lesion volume (lesion load) in each patient, an expert neuroradiologist with more than 10 years of experience segmented the WM lesions in the MS group by manually adding contours to the 3D-T2-weighted FLAIR images using the BRAIM program.

### 2.4. Voxel-Based Morphometry Analysis (VBM)

We used SPM8 software for the VBM analysis. Pre-processing steps included origin setting at the anterior commissure, GM and WM segmentation, normalization with the DARTEL toolbox, and smoothing with an 8-mm full-width-at-half-maximum Gaussian. To identify brain regions with significant group differences in GM volume, the GM maps were statistically analyzed using a two-sample *t*-test. Age, gender, and ICV were included as nuisance covariates. Statistical maps were FDR corrected (*p* < 0.05) for multiple comparisons at the voxel level.

To investigate the association between regional GM volumes and each score or sub-score obtained in the cognitive testing, multiple regression analyses were performed with age, gender, and ICV as covariates of no interest. A statistical threshold of *p* < 0.001 (uncorrected) and an extent threshold of 20 voxels were used.

### 2.5. DTI Analysis

The diffusion-weighted data were analyzed with FSL v5.0 (FMRIB, Oxford, UK.). The preprocessing steps included head motion correction, correction for eddy-current distortion, and diffusion tensor fitting with FMRIB’s Diffusion Toolbox (FDT v 3.0). The tract-based spatial statistics program (TBSS) was used for the voxel-wise statistical analysis of the DTI data. The mean fractional anisotropy (FA) skeleton was computed after the FA map registration, and aligned to the average space as input for TBSS. Voxel-wise statistics were performed using the randomize tool with 5000 permutations and a threshold of 0.2, with age, gender, and ICV as covariates. Family-wise error-corrected maps were obtained with *p* < 0.05.

After the TBSS analysis, the WM regions that were significantly different in the MS group were localized using a probabilistic digital atlas of the major WM tracts provided by FSL [27] and selected for tractography. For tractography, pre-processing, and the generation of fiber tracts, the data were processed using ExploreDTI [28]. After eddy-current and head-motion correction, the DTI was estimated using a non-linear least square approach [29]. DTI scalar maps, including FA and mean diffusivity (MD), were calculated. Whole-brain tractography was performed by selecting all seed voxels with an FA > 0.2. Streamlines were propagated using Euler integration [30] and a tractography algorithm step size of 1 mm. The whole-brain tractography data were imported into TrackVis software v.0.6 [31] for virtual dissection of the tracts. The tracts were dissected using the two-regions-of-interest approach based on a previously published atlas [32,33]. Based on the TBSS results, virtual dissections of the corpus callosum, fornix, both corticospinal tracts, cingula, uncinate fasciculi, inferior longitudinal fasciculi, and inferior fronto-occipital fasciculi were performed. For each participant, the corpus callosum (CC) was manually segmented into the seven subdivisions defined by Witelson [34], (orbitofrontal, anterior frontal, superior frontal, superior parietal, posterior parietal, temporal, and occipital), and consistent with previous studies [35,36,37].

The connectivity-based thalamic parcellation was assessed using FSL v.5.0. Cortical masks (frontal, parietal, temporal, and occipital) were obtained from the Harvard Oxford subcortical and MNI structural atlas in FSL. Masks were thresholded to exclude the WM and then binarized. The segmented thalami from each participant were used to form the thalamic mask. DTI scalar maps were registered to the standard space with FMRIB’s FLIRT linear registration tool, and FA and MD values were obtained for the whole thalami (each hemisphere separately). Thalamocortical connections were assessed using a connectivity-based seed classification with the distance correction tool in ProbtrackX [38]. Each thalamic voxel was thresholded to include only those projections with a probability of ≥50% and then binarized. For each thalamocortical connection, the MD values and the volumes normalized to the ICV were determined, as previously described [39].

### 2.6. Thalamic Segmentation and Vertex Analysis

Automated segmentation of the thalamus was performed using FIRST (FMRIB’s integrated registration and segmentation tool) [40]. A vertex analysis was performed on the FIRST output to show thalamic shape changes. Regional changes in the vertices across groups were assessed using a global linear model. The results were corrected for multiple comparisons using FDR values (*p* < 0.05). The statistic was rendered on the surface, providing a detailed map of where the structure changed significantly between groups.

### 2.7. Statistical Analysis of Quantitative Variables

We used SPSS software (SPSS 20.0). Age and sex differences between groups were assessed by a t-test and chi-square test, respectively. Quantitative variables are expressed as mean ± standard or median with interquartile range, as appropriate. The Kolmogorov–Smirnov test was used to determine the normality of the distributions of the variables. Demographic and/or imaging metrics were compared between groups (patients *vs*. controls) using the Mann–Whitney *U* test or unpaired student *t*-test, as appropriate.

For correlation studies, the Pearson rank correlation or the Spearman rank correlation tests were used as appropriate. In all cases, *p* < 0.05 was considered to indicate statistical significance.

## 3. Results

### 3.1. Demographic Characteristics and Cognitive Performance

The study included 30 patients with RRMS and 30 age-sex matched healthy controls (9 men and 21 women). Most patients (*n* = 28/30) were treated with the following: interferon-beta (*n* = 14), dimethyl fumarate (*n* = 5), glatiramer (*n* = 5), natalizumab (*n* = 3), and fingolimod (*n* = 1). The mean disease duration was 9.5 ± 6.3 years and the median EDSS was 2 (range: 0–3). The demographic characteristics, MRI data, and cognitive performances of the patients and controls are summarized in Table 1.

Patients with MS scored significantly lower than healthy controls on the SDMT. No significant between-group differences were observed in the global M@T scores. However, when analyzing the M@T subtest scores, the MS group showed significantly lower scores in subtests of free recall, cued-recall, and episodic memory. No statistically significant intergroup differences were observed in the temporal orientation or semantic memory subtests.

### 3.2. Neuroimaging Analyses

#### 3.2.1. Segmentation of Brain and Subcortical Structures

WM, GM, and BPF volumes were significantly lower in the MS group than in the control group (Table 1). Segmentation of the subcortical structures showed significant volume loss affecting the thalamus, putamen, caudate nucleus, and hippocampus bilaterally. No significant interhemispheric differences were observed in the segmented subcortical structures in either group.

#### 3.2.2. VBM

**Figure 1:**
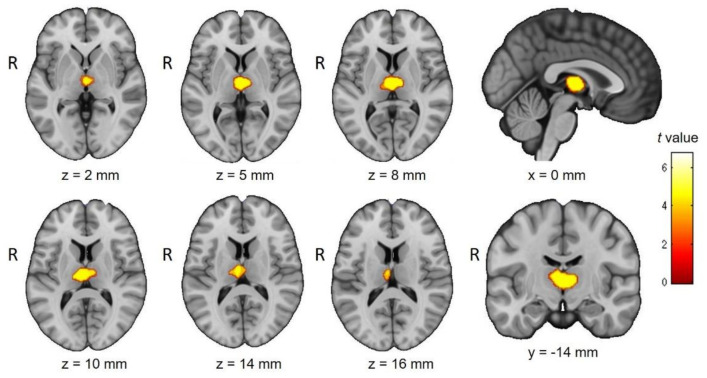
Between-group differences in gray matter (GM) volumes. Yellow clusters reflect GM reductions in the MS group affecting both thalami. The color bar represents the *t*-score. Significance is indicated by *p* < 0.05, FDR corrected; extent threshold = 10 voxels.

Patients with MS showed a significant reduction in GM in both thalami. In contrast, the reverse comparison (MS > controls) yielded no significant voxels, indicating that MS participants did not have more regional GM in any brain region compared to controls (Figure 1).

**Figure 2:**
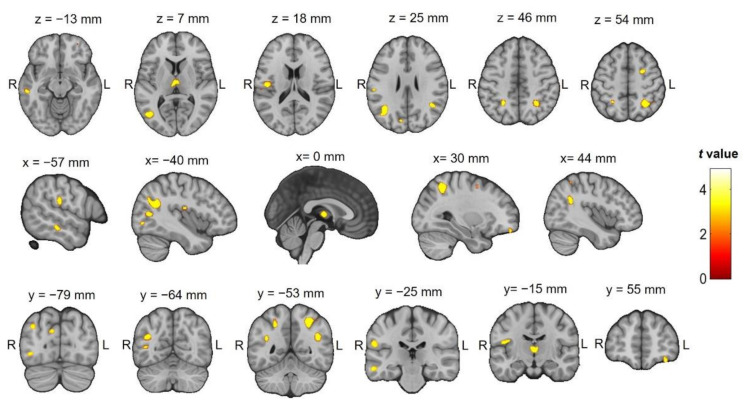
Multiple regression analysis using voxel-based morphometry to identify the GM regions significantly correlated with scores on the symbol digit modalities test (SDMT), a test of cognitive impairment, in the MS group. The color bar represents the *t*-score. Significance is indicated by an uncorrected *p* < 0.001; extent threshold = 20 voxels.

Multiple regression analyses within the MS group revealed an association between voxel clusters showing GM loss and SDMT scores affecting both hemispheres, specifically the left middle temporal gyrus, right superior temporal gyrus, left postcentral gyrus, right inferior and superior parietal lobules, the medial dorsal nucleus of the thalamus bilaterally, the right frontal superior gyrus, the left insula, the left inferior occipital gyrus, the cuneus, and the right frontal lobe at the cingulate gyrus (see Figure 2 and Table 2). No significant associations were observed between the T@M global or subtest scores and GM volume.

#### 3.2.3. Diffusion Tensor Imaging

**Figure 3:**
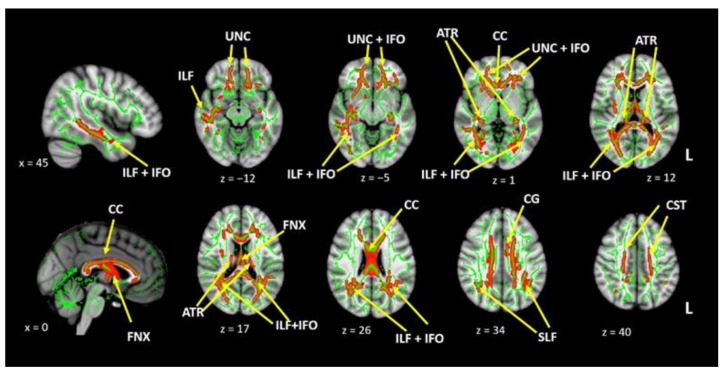
Tract-based spatial statistics (TBSS) results showing differences in fractional anisotropy (FA) between patients with MS and controls. The red-yellow scale indicates areas with reduced FA values in the MS group. The thresholded statistical image has been thickened for clarity. UNC: uncinate fasciculus, IFO: inferior fronto-occipital fasciculus, ILF: inferior longitudinal fasciculus, CC: corpus callosum, FNX: fornix, CST: corticospinal tract, CG: cingulum, ATR: anterior thalamic radiation.

In the MS group, the TBSS results showed significantly lower FA values (corrected for multiple comparisons) in the corpus callosum (CC), fornix (FNX), both uncinate fasciculi (UF), both inferior longitudinal (ILF) and fronto-occipital fasciculi (IFO), both cingula (CG), and both corticospinal tracts (CST) (see Figure 3). We observed no increases in FA in the MS group compared to controls.

**Figure 4:**
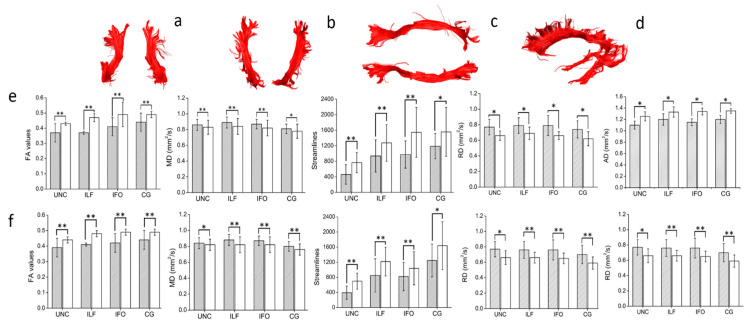
An illustrative reconstruction of the association of white matter (WM) tracts in a healthy control participant. (**a**) Uncinate fasciculi: UNC, (**b**) inferior longitudinal fasciculi: ILF, (**c**) inferior fronto-occipital fasciculi: IFO, (**d**) cingula: CG. (**e**,**f**) Diffusion tensor metrics for WM tracts for the (**e**) left hemisphere, and (**f**) right hemisphere. Filled bars represents MS results; white bars represents controls results. Bars are mean ± SD. * *p* < 0.05; ** *p* < 0.005; all unpaired two-tailed comparisons between MS and controls.

**Figure 5:**
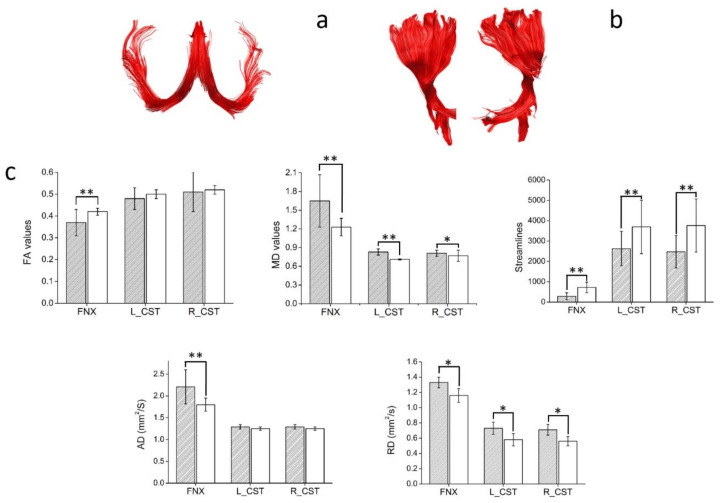
An illustrative reconstruction of the projection WM tracts in a control participant. (**a**) fornix: FNX, (**b**) corticospinal tracts: CST. (**c**) Diffusion tensor metrics. Patients’ results are shown as filled bars,; controls results are shown as white bars. L_CST: left corticospinal tract, R_CST: right corticospinal tract. Bars are mean ± SD. * *p* < 0.05; ** *p* < 0.005; all unpaired two-tailed comparisons between patients with MS and controls.

**Figure 6:**
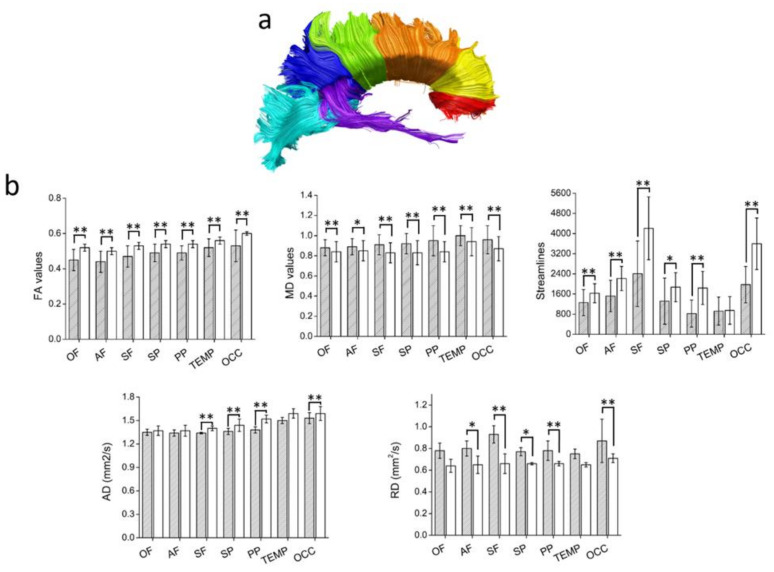
(**a**) An illustrative reconstruction of the CC in a control participant, with colors representing a seven-way tractography segmentation according to a probabilistic atlas of the CC. The segments are as follows: orbital frontal division (OF, red), anterior frontal (AF, yellow), superior frontal (SF, orange), superior parietal (SP, green), posterior parietal (PP, dark blue), temporal (TEMP, purple), and occipital (OCC, light blue). (**b**) Diffusion tensor metrics. Patients’ results are shown as filled bars; controls are shown as white bars. Bars are mean ± SD. * *p* < 0.05; ** *p* < 0.005; all unpaired two-tailed comparisons between patients with MS and controls.

Manual dissections of the WM tracts revealed statistically significant differences in the diffusion metrics of all the WM fascicles analyzed (see Figure 4, Figure 5 and Figure 6).

#### 3.2.4. Vertex Analysis and Thalamic Parcellation

**Figure 7:**
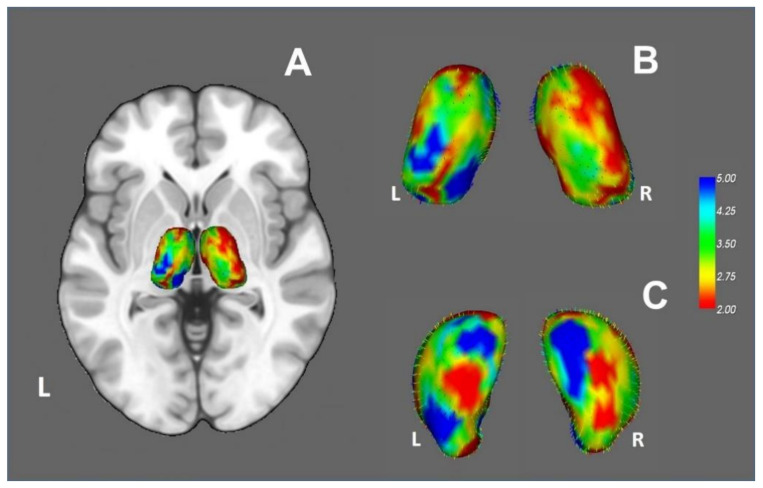
(**A**) Shape analysis of the left and right thalami in MNI space. Both thalami showed significant changes in the MS group, suggesting volume loss. Blue represents the regions of the thalamus that showed the most significant changes. (**B**,**C**) A magnified view of the shape analysis, with vectors representing the direction of the shape change (most vector arrowheads cannot be visualized because they point inward). (**C**) A view rotated to show the inferior surfaces of the thalami.

Group comparison revealed significant changes in the thalamus shape in the MS group, suggesting volume loss and therefore atrophy (see Figure 7). The greatest changes were observed in the anterior and medial aspects of the thalami bilaterally.

We observed statistically significant differences in the FA and MD values of all thalamocortical connections (Table 3), except for the MD values of the right hemisphere thalamic connections to the frontal cortex. Moreover, significant lower whole-thalamus FA values in patients with MS, as well as higher whole-left-thalamus MD values, were observed.

#### 3.2.5. Correlations between MRI Measurements, Disease Severity, and Cognitive Performance

We conducted a series of correlation analyses to assess possible relationships among MRI measurements, disease severity (EDSS) scores, and cognitive performance scores.

For GM volume, thalamic volume was correlated with EDSS (r = −0.4, *p* = 0.02) and SDMT scores (r = 0.5, *p* = 0.005). A negative correlation was observed between disease duration in years and GM volumes and between disease duration and hippocampus volumes (r = −0.4, *p* = 0.02; r = −0.43, *p* = 0.01, respectively). BPF was negatively correlated with EDSS (r = −0.47, *p* = 0.009). No other significant correlations were observed.

For WM tracts, EDSS was negatively correlated with the CC streamlines, specifically with the posterior parietal and superior parietal projection fibers (Table 4) and the fornix streamlines. SDMT scores were correlated with the streamlines at the occipital projection fibers of the CC and the right inferior fronto-occipital fasciculus. A positive correlation was observed between the left uncinate streamlines and the M@T subtests (encoding, free recall, cued-recall, and episodic). The left inferior longitudinal fasciculus streamlines were correlated with the M@T free recall, cued-recall, and episodic subtests. Finally, the right cingulum and CC streamlines were correlated with encoding and episodic subtests.

We observed a negative correlation between the EDSS and SDMT results and the FA values of all the thalamic connections evaluated, and a positive correlation with the MD values for all thalamic connections, except for the right occipital (Table 5).

## 4. Discussion

This study investigated whether damage to specific WM tracts and gray matter areas could be responsible for the CI observed in mildly disabled RRMS patients. Many studies have analyzed CI in MS as a binary condition: present or not. However, we considered that CI should be seen as a continuum. We thus evaluated cognitive performance as cognitive processing speed and episodic memory in patients and controls. The SDMT was used to evaluate cognitive processing speed because it is considered the task most sensitive to MS and is highly recommended as a cognitive monitoring tool in clinical practice [41]. Instead of BICAMS [42] for episodic memory, we used the M@T, a verbal episodic and semantic memory test that has been validated in primary care populations in Spain [24].

In the MS group, significant volume loss affecting the WM, GM, and subcortical structures was observed. However, only thalamic volume was associated with the EDSS and SDMT scores. Our results are consistent with those of previous studies that identified thalamic atrophy as the main MRI marker associated with CI [43]. In addition, our results pointed to compromised WM integrity in all thalamocortical projections, which in turn associated with the EDSS and SDMT scores. As we studied patients with mildly disabling RRMS and low macroscopic lesion loads on MRI images, our results are consistent with those of a previous study by Deppe et al., who found significant thalamic atrophy in the early phase of the disease [44]. Such changes may be caused by microstructural destructive processes within the thalamus itself rather than by retrograde neuroaxonal degeneration and/or anterograde transsynaptic changes secondary to WM lesions, without GM involvement. In this sense, our results are consistent with those of previous reports, suggesting that a worsening disability as assessed by the EDSS is related to thalamic damage and subsequent thalamocortical disconnection [45,46,47].

We also found significant associations between specific Brodmann areas and SDMT scores. Specifically, we found associations with GM volume at both the angular and supramarginal gyri (BA39 and BA40, respectively). These areas integrate various input modalities (somatosensory, visual, and auditory) that play an important role in several higher cognitive functions (i.e., episodic and semantic memory, mathematical abilities, literacy, and spatial attention) [48]. They also have connections to regions such as the frontal lobe through the superior longitudinal fasciculus, to the caudate nucleus through the inferior fronto-occipital fasciculus, and to the hippocampal gyrus through the inferior longitudinal fasciculus. Moreover, we found associations with motor coordination areas BA6 and BA7, visual areas BA18 and BA19, language processing area BA21, and executive area BA10. Overall, our results suggest that SDMT performance may be related to the integrity of specific brain areas that are involved in visual-spatial processing, manual coordination, language processing, and higher executive functions. Characteristically, these areas are related to the frontoparietal attention network, whose inputs are processed in the thalamus, which acts as a relay to, or between, cortical regions [49]. This thalamic control function is critical for functional cortical networks, impacting how cognitive processes such as attentional control unfold over cortical space and time. Thus, the observed thalamic atrophy, alongside the extensive impairment to the thalamocortical projections observed in our cohort, may have impaired the coordination of the information processed in these areas, leading to reduced SDMT performance. Furthermore, we found an association between the SDMT and the streamlines at the right IFO. This associative fasciculus connects the occipital cortex, basal temporal lobe, superior parietal lobe, and frontal lobe [50]. Despite persistent controversy concerning its function, several studies have proposed that the IFO has an essential function in semantic processing and language [51], visual conceptualization, and recognition [52]; functions which are required to perform the SDMT test correctly. In a group of patients with cerebral small-vessel disease and WM hyperintensities, Chen et al. found that damage to the right IFO was associated with poor performance in the MMSE test, suggesting that this bundle may be used as an imaging marker for early recognition of WM-related cognitive impairment [53]. Given the size, length, and inherent multi-site connectivity of this tract, a reduction in the streamlines in this fascicle may affect the coordination and processing of information flowing between these areas, thereby affecting performance on the SDMT.

In our study, segmentation of the CC into seven portions allowed for a more detailed localization of damage and association to clinical and cognitive variables. As the main interhemispheric commissure, the CC provides an interaction between the cerebral hemispheres and plays a role in complex cognitive tasks. In MS, the CC is often affected by demyelinating lesions, and atrophy of this structure is very common. Our results are consistent with those of histopathological studies showing axon loss and decreased fiber density in the CC of participants with MS [54]. However, despite the widespread damage observed in the CC, only changes in the occipital projection fibers, related to visual and auditory stimuli, were correlated with the SDMT scores. We found that the total streamlines at the CC were associated with some memory subtest scores, namely encoding, cued-recall, and episodic, supporting previous studies suggesting that the structural disconnection of the CC due to axonal damage may contribute, at least partially, to the development of CI, likely through a multiple disconnection syndrome [15,55,56].

In our study, only the episodic memory subtest scores were correlated with WM measurements, suggesting that WM damage plays a more important role in impairing episodic memory than GM damage. In this regard, we found associations between the streamlines at the left uncinate and left inferior longitudinal fasciculus, and memory subtest scores. Both fascicles are part of the anterior temporal network, have direct contact, and project into the anterior temporal structures. They provide an indirect anatomical connection between the posterior temporal, occipital, and frontal areas, forming the ventral stream for language and semantic processing [57]. Although the function of the uncinate fasciculus remains unclear, it has been associated with several functions such as episodic memory and language (i.e., proper naming, semantic retrieval) [58]. In addition, it is important to note that the uncinate is one of the last fasciculi to myelinate in the adult brain, at around the third decade of life, rendering it more vulnerable to damage if the disease occurs before its full myelination [59]. Regarding the ILF, this long bundle connects the temporal and occipital lobes; while some controversy remains concerning its function. Recent studies have shown that disruptions of this tract are associated with cognitive disorders, especially in the left hemisphere, as well as impairments in lexical retrieval and naming [57]. Finally, we found an association between the streamlines at the right cingulum and episodic and encoding memory. As an associative tract that interconnects the frontal, parietal, and medial temporal lobes with subcortical nuclei, the cingulum plays a critical role in cognitive control [60]. Our observations complement those of previous studies suggesting that lesions in the cingulum are related to problems with episodic memory [61], verbal memory [62], and language [63].

Consistent with previous studies, our data pointed to the presence of a fornix injury in the MS group, independently of the lesion load and volumetric results. This observation was associated with episodic memory, supporting previous studies on fornix pathology [64]. Similar results have been found in patients with MS, independent of clinical subtype, suggesting that this injury could be secondary to the observed hippocampal atrophy [65].

Several limitations of the study must be considered. First, the results reported were derived from one population and have not been independently tested. Since this is a case-control study from one institution, additional studies are necessary for confirmation. Second, the sample size was relatively small. However, we were able to uncover statistically significant comparisons and correlations, which supports the validity of the data. Third, since the study consisted only of patients with RRMS, the results are not valid for patients with progressive MS. Fourth, the cross-sectional design prevented us from drawing any conclusions about temporal relationships between the observed GM and WM damage in our cohort.

Overall, our results are consistent with those of previous studies, suggesting that patients with MS with mildly disabling RRMS and low lesion loads on MRI may already present significant WM damage and GM volume loss. Our observations agree with previous reports on MS, suggesting that CI in MS may arise from a combination of WM and GM damage, whereby several WM tracts and functional domains are affected, leading to a wide range of clinical manifestations.

## 5. Conclusions

In summary, our results suggest that in patients with RRMS and mild disease, thalamic atrophy and damage to the thalamocortical connections are associated with slower cognitive processing, and WM damage at specific fasciculi is related to episodic memory impairment.

## Figures and Tables

**Table 1:** Main demographic, clinical, and MRI characteristics of the subjects enrolled in the study.

	MS (*n* = 30)	HC (*n* = 30)	*p*
Sex (M/F) (number)	(21/9)	(21/9)	0.83
^†^ Age years (range)	41 (24–63)	40 (22–60)	0.71
^‖^ Disease duration years (SD)	9.5 ± 6.3	*n.a.*	
^†^ Education years	13 (9–17)	15; 4 (9–17)	0.04
^†^ EDSS	2 (0–2.5)	*n.a*.	
^‖^ z-SDMT scores (SD)	41.31 ± 11.25	56.56 ± 9.44	<0.001
^‖^ z-M@T global score (SD)	48.18 ± 5.5	48.55 ± 5.3	0.799
^‖^ z-M@T-encoding	8.93 ± 1.1	9.81 ± 0.4	<0.001
^‖^ z-M@T-orientation	5.24 ± 1.7	4.96 ± 0.19	0.97
^‖^ z-M@T-semantic	13.55 ± 3.05	14.50 ± 0.58	0.29
^‖^ z-M@T-free recall	7.59 ± 1.8	9.12 ± 0.86	0.001
^‖^ z-M@T-cued-recall	8.76 ± 1.52	9.7 ± 0.54	0.003
^‖^ z-M@T-episodic **	16.3 ± 3.15	19 ± 0.95	<0.001
^‖^ GMV (mL)	633.4 ± 104.2	717.64 ± 87.2	0.002
^‖^ WMV (mL)	399.91 ± 63.52	453.82 ± 61.8	0.002
^‖^ CSFV (mL)	493.29 ± 111.9	469.87 ± 85.34	0.374
^‖^ Putamen (mL)	4.06 ± 0.82	4.47 ± 0.36	0.017
^‖^ Thalami (mL)	9.51 ± 1.72	10.32 ± 1.23	0.01
^‖^ Hippocampus (mL)	3.89 ± 0.65	4.82 ± 0.54	<0.001
^‖^ Caudate (mL)	4.11 ± 0.7	5.03 ± 0.56	<0.001
^‖^ ICV (mL)	1526.6 ± 159	1641.33 ± 164.3	0.009
^‖^ BPF	0.67 ± 0.6	0.71 ± 0.38	0.008
^‖^ WM lesion load (mL)	9.55 ± 4.53	*n.a.*	

MS: multiple sclerosis patients, HC: healthy controls, M: male, F: female, *n.a*.: not applicable, ** free recall plus cued-recall subtests, GMV: grey matter volume, WMV: white matter volume, CSFV: cerebrospinal fluid volume, ICV: intra-cranial volume, BPF: brain parenchymal fraction, EDSS: expanded disability status scale. ^†^ Data are median; interquartile range. ^‖^ Data are means ± standard deviation. *p* values are comparisons between patient and control groups.

**Table 2:** Regions of positive correlations between regional gray matter volumes and SDMT z-scores in MS patients.

Brain Area	Hemisphere	BA	x	y	z	Z Value	Cluster
Temporal Lobe, Middle Temporal Gyrus	L	39	−37	−63	25	4.89	420
Parietal Lobe, Superior Parietal Lobule	R	7	30	−55	49	4.72	370
Temporal Lobe, Superior Temporal Gyrus	R	39	43	−52	28	4.28	137
Temporal Lobe, Middle Temporal Gyrus	L	21	−57	−27	−13	4.17	68
Thalamus, Medial Dorsal Nucleus	L		−4	−13	6	4.05	174
Thalamus, Medial Dorsal Nucleus	R		6	−13	6	3.94	
Insula	L	13	−46	−16	18	3.90	106
Temporal Lobe, Middle Temporal Gyrus	L	39	−43	−69	10	3.9	229
Parietal Lobe, Sub-Gyral	L	7	−25	−49	52	3.89	106
Parietal Lobe, Postcentral Gyrus	L	40	−58	−24	22	3.84	87
Frontal Lobe, Sub-Gyral cingulate	R	6	27	2	52	3.78	85
Occipital Lobe, Cuneus	L	18	−10	−78	27	3.74	22
Occipital Lobe, Inferior Occipital Gyrus	L	19	−41	−79	−4	3.67	57
Frontal Lobe, Superior Frontal Gyrus	R	10	−36	−79	33	3.41	33
Parietal Lobe, Inferior Parietal Lobule	R	40	42	-49	55	3.37	21

Cluster size is expressed in number of voxels at *p* < 0.001 uncorrected. BA: Brodmann area, L: left, R: right.

**Table 3:** Mean and standard deviations of FA values and MD (mm^2^/s) values within the whole thalamus and the four segmented thalamocortical projections. *p* values are unpaired two-tailed test comparisons between MS patients and control (HC) groups.

	MS	HC	*p*
L-Thalamus FA	0.22 ± 0.012	0.25 ± 0.04	<0.001
L-Thalamus MD	0.0011 ± 0.00009	0.001 ± 0.00005	<0.001
R-Thalamus FA	0.21 ± 0.012	0.25 ± 0.03	<0.001
R-Thalamus MD	0.0014 ± 0.0018	0.0017 ± 0.0015	0.65
L-Frontal FA	0.28 ± 0.02	0.34 ± 0.016	<0.001
L-Frontal MD	0.0015 ± 0.00023	0.0013 ± 0.00011	<0.001
R-Frontal FA	0.29 ± 0.03	0.33 ± 0.02	<0.001
R-Frontal MD	0.0013 ± 0.00027	0.0013 ± 0.00016	0.87
L-Parietal FA	0.28 ± 0.02	0.34 ± 0.016	<0.001
L- Parietal MD	0.0014 ± 0.0002	0.001 ± 0.0001	<0.001
R- Parietal FA	0.29 ± 0.03	0.33 ± 0.02	<0.001
R- Parietal MD	0.0013 ± 0.0003	0.001 ± 0.0001	<0.001
L-Temporal FA	0.28 ± 0.02	0.34 ± 0.01	<0.001
L- Temporal MD	0.0013 ± 0.0002	0.001 ± 0.00001	<0.001
R- Temporal FA	0.29 ± 0.03	0.33 ± 0.02	<0.001
R- Temporal MD	0.0013 ± 0.0003	0.001 ± 0.0001	<0.001
L-Occipital FA	0.28 ± 0.02	0.33 ± 0.016	<0.001
L- Occipital MD	0.0013 ± 0.0002	0.001 ± 0.0001	<0.001
R- Occipital FA	0.29 ± 0.03	0.33 ± 0.02	<0.001
R- Occipital MD	0.0015 ± 0.0013	0.001 ± 0.0001	0.03

L: left, R: right. L-frontal and R-frontal: thalamic projections connected to the frontal cortex; L-parietal and R-parietal: thalamic projections connected to the parietal cortex; L-temporal and R-temporal: thalamic projections connected to the temporal cortex; L-occipital and R-occipital: thalamic projections connected to the occipital cortex.

**Table 4:** Correlation results between clinical, cognitive tests, and tractography results. Only the significant results are shown.

	EDSS		z-SDMT		M@T-Encoding		M@T-Free Recall		M@T-Cued Recall		M@T-Episodic	
	*r*	*p*	*r*	*p*	*r*	*p*	*r*	*p*	*r*	*p*	*r*	*p*
CC STR	−0.4	0.03	*ns*	*ns*	0.64	<0.001	*ns*	*ns*	−0.58	0.001	0.5	0.007
CC-Superior parietal STR	−0.48	0.009	*ns*	*ns*	*ns*	*ns*	*ns*	*ns*	*ns*	*ns*	*ns*	*ns*
CC-Superior parietal AD	0.62	<0.001	*ns*	*ns*	*ns*	*ns*	*ns*	*ns*	*ns*	*ns*	*ns*	*ns*
CC-Posterior Parietal STR	−0.41	0.02	*ns*	*ns*	*ns*	*ns*	*ns*	*ns*	*ns*	*ns*	*ns*	*ns*
CC-Posterior Parietal AD	0.45	0.01	*ns*	*ns*	*ns*	*ns*	*ns*	*ns*	*ns*	*ns*	*ns*	*ns*
CC-Occipital STR	ns	ns	0.41	0.027	*ns*	*ns*	*ns*	*ns*	*ns*	*ns*	*ns*	*ns*
FNX STR	−0.52	0.004	*ns*	*ns*	*ns*	*ns*	*ns*	*ns*	*ns*	*ns*	*ns*	*ns*
FNX FA	*ns*	*ns*	*ns*	*ns*	*ns*	*ns*	*ns*	*ns*	*ns*	*ns*	0.4	0.04
FNX AD	0.55	0.002	*ns*	*ns*	*ns*	*ns*	*ns*	*ns*	*ns*	*ns*	0.43	0.02
L-UNC STR	*ns*	*ns*	*ns*	*ns*	0.56	0.001	0.62	<0.001	0.43	0.018	0.57	0.001
L-UNC AD	*ns*	*ns*	*ns*	*ns*	0.61	<0.001	*ns*	*ns*	*ns*	*ns*	*ns*	*ns*
L-ILF STR	*ns*	*ns*	*ns*	*ns*	*ns*	*ns*	0.51	0.004	0.37	0.04	0.477	0.009
R-IFO STR	*ns*	*ns*	0.46	0.012	*ns*	*ns*	*ns*	*ns*	*ns*	*ns*	*ns*	*ns*
R-IFO AD	*ns*	*ns*	−0.47	0.01	*ns*	*ns*	*ns*	*ns*	*ns*	*ns*	*ns*	*ns*
R-CG STR	*ns*	*ns*	*ns*	*ns*	0.54	0.002	*ns*	*ns*	*ns*	*ns*	0.38	0.04
R-CG AD	*ns*	*ns*	*ns*	*ns*	0.51	0.005	*ns*	*ns*	*ns*	*ns*	*ns*	*ns*
L-CST AD	0.39	0.03	*ns*	*ns*	*ns*	*ns*	*ns*	*ns*	*ns*	*ns*	*ns*	*ns*
R-CST AD	0.56	0.001	*ns*	*ns*	*ns*	*ns*	*ns*	*ns*	*ns*	*ns*	*ns*	*ns*

CC: corpus callosum, STR: streamlines, FNX: fornix, UNC: uncinate fasciculus, ILF: inferior longitudinal fasciculus, IFO: inferior fronto-occipital fasciculus, CG: cingulum, L: left hemisphere, R: right hemisphere, *ns*: not significant.

**Table 5:** Correlation results for MS patients between the EDSS and SDMT tests with the segmented thalamocortical connections. Only the significant results are shown.

	EDSS		z-SDMT	
	*r*	*p*	*r*	*p*
L-Thalamus FA	−0.65	<0.001	0.4	0.03
L-Thalamus MD	0.7	<0.001	*ns*	*ns*
R-Thalamus FA	−0.65	<0.001	*ns*	*ns*
R-Thalamus MD	*ns*	*ns*	*ns*	*ns*
L- frontal lobe FA	−0.4	0.03	−0.4	0.03
L- frontal lobe MD	0.55	0.002	0.55	0.002
L- parietal lobe FA	−0.44	0.01	−0.44	0.01
L- parietal lobe MD	0.58	<0.001	0.58	<0.001
L-Temporal FA	−0.44	0.016	−0.44	0.01
L-Temporal Lobe MD	0.55	0.002	0.55	0.002
L-Occipital lobe FA	−0.41	0.02	−0.41	0.02
L-Occipital lobe MD	0.52	0.003	0.52	0.003
R- frontal lobe FA	−0.53	0.003	−0.53	0.001
R- frontal lobe MD	0.56	0.001	0.56	0.002
R-Parietal Lobe FA	−0.52	0.003	−0.52	0.003
R-Parietal Lobe MD	0.55	0.001	0.55	0.001
R-Temporal lobe FA	−0.52	0.003	−0.53	0.003
R-Temporal lobe MD	0.57	0.001	0.57	0.001
R-occipital lobe FA	−0.52	0.003	−0.52	0.003
R-occipital lobe MD	*ns*	*ns*	*ns*	*ns*

L: left hemisphere, R: right hemisphere, *ns*: not significant.

## Data Availability

The data presented in this study are not publicly available due to privacy issues.
